# Les urgences infectieuses ORL

**DOI:** 10.11604/pamj.2016.25.27.9830

**Published:** 2016-09-27

**Authors:** Moustapha Sereme, Souleymane Tarnagda, Patrice Guiguimde, Yvette Marie Chantal Gyebre, Bertin Ouedraogo, Bambara Céline, Maimouna Ouattara, Kampadilemba Ouoba

**Affiliations:** 1 Service d’ORL et de Chirurgie Cervico-Faciale du CHU Yalgado Ouédraogo, Burkina Faso; 2 Service d’Odontostomatologie et de Chirurgie Maxillo-Faciale de CHU Yalgado Ouédraogo, Burkina Faso

**Keywords:** Urgences, infectieuses, ORL, sous médicalisation, pauvreté, Emergencies, infectious, ENT, undermedicalization, poverty

## Abstract

Affections gravissimes à pronostic très réservé particulièrement dans notre contexte de sous médicalisation et de pauvreté. Notre but en initiant ce travail est de déterminer les étiologies de ces urgences et discuter de leur prise en charge thérapeutique. Étude de type rétrospective et descriptive sur 05 ans, au total 52 dossiers cliniques ont été inclus. Ces infections ont représenté 0,33% de nos consultations. La moyenne d'âge de nos patients a été 23 ans. Le jeune âge, les traitements inappropriés et certaines affections ORL ont été retrouvés comme facteurs favorisants. Les motifs de consultation ont été variés en fonction du siège de l'infection, cependant deux signes cliniques ont été constants: la douleur et la fièvre. L'adénophlegmon, le phlegmon péri-amygdalien, les cellulites ont été nos principales étiologies avec le streptocoque et le staphylocoque comme principaux germes en cause. L'antibiothérapie probabiliste a été utilisée en première intention notamment l'association céphalosporine de 3ème génération + aminoside + imidazolé. L'évolution clinique de nos patients a été marquée par la survenue de complications locales et générales. Urgence diagnostic et thérapeutique leur évolution reste encore émaillée de complication en raison de la consultation tardive de nos patients.

## Abstract (en)

Extremely serious diseases associated with very poor prognosis, especially in this context of undermedicalization and poverty. The aim of this case study was to determine the etiologies of these emergencies and to discuss their therapeutic management. Retrospective-descriptive study conducted over a 5-year period, a total of 52 clinical records were included. These infections accounted for 0.33% of our consultations. The average age of our patients was 23 years. Young age, inappropriate treatments and some ENT disorders were found to be contributing factors. The reasons for consultation were varied, depending on the site of infection; however, two clinical signs were constant: pain and fever. Adenophlegmon, peritonsillar phlegmon, cellulitis were our main etiologies together with streptococcus and staphylococcus which were the commonest causative bacteria. Empirical antibiotic therapy was used as first-line therapy, in particular third-generation cephalosporin + aminoglycoside + imidazole combination. Clinical course was marked by local and systemic complications. The evolution of diagnostic and therapeutic ENT emergencies management plans still encounters complications due to patient delay in seeking consultation.

## Introduction

Les infections ORL sont fréquentes en consultation médicale courante en effet selon la Société de Pathologie Infectieuse de Langue Française ( SPILF) ces infections ORL sont responsable de plus de la moitié des prescriptions d'antibiotiques [1]. Dans la littérature la fréquence des urgences infectieuses est variable d'un auteur à l'autre [1–3]. Cependant, l'ensemble de ces auteurs affirment le caractère angoissant de ces urgences en raison de leur pronostic parfois réservé. Notre étude se donne comme objectif de déterminer les causes des urgences infectieuses ORL dans notre contexte d'exercice et de discuter de la conduite à tenir.

## Méthodes

Il s'est agi d'une étude de type rétrospective menée du 1^er^ Janvier 2010 au 31 Décembre 2014 dans le service d'ORL et de chirurgie cervico-faciale du CHU Yalgado Ouédraogo. Il a été inclus dans l'étude les patients pris en charge en urgence pour une pathologie ORL et qui avaient un dossier clinique complet soit 52 dossiers. Il a été considéré comme urgence infectieuse, toutes infections ORL mettant en jeu de façon immédiate le pronostic vital du patient. Les paramètres pris en compte dans l'étude ont été épidémiologiques (fréquence, âge de survenue, sex-ratio, facteurs favorisants), clinique et paracliniques (les délais de consultation, les signes d'appels, les résultats de l'examen physique et des examens paracliniques), thérapeutiques (familles de médicaments utilisés, gestes chirurgicaux pratiqués, les indications thérapeutiques) et évolutifs.

## Résultats

### Données épidémiologiques

**Fréquence:** durant notre période d'étude (Janvier 2010-Décembre 2014), 15750 patients ont consulté en ORL dont 52 cas d'urgences infectieuses. Ces urgences ont ainsi représenté 0,33% de nos consultations. Leur incidence annuelle a été de 5,2 cas.

**Sexe:** notre collectif a été constitué de 26 patients de sexe masculin et 26 de sexe féminin. Le sex-ratio a été de 1.

**Age:** La moyenne d'âge de nos patients a été 23 ans avec des extrêmes de 5 mois et de 76 ans. La tranche d'âge de 0 à 10 ans a été la plus représentée, nous notons cependant une augmentation de la fréquence de ces urgences entre 60 et 70 ans.

**Niveau socio-économique:** Les élèves et les étudiants ont été la catégorie socio-professionnelle la plus rencontrée suivie des femmes au foyer (voir Tableau 1).

**Tableau 1:** Catégorie socio-professionnelle de nos patients

Catégorie socio-professionnelle	Nombre	Pourcentage
Elèves et étudiants	17	32,5
Femmes au foyer	12	23
Enfants non scolarisés	07	13,5
Secteur informel (chauffeurs, commerçants	07	13,5
Sans emploi	05	9,5
Cultivateurs	02	4
Fonctionnaires	02	4
**Total**	**52**	**100**

**Facteurs favorisants:** Le jeune âge : 27% de nos patients ont moins de 10 ans et 48% moins de 20 ans.

*Certaines infections ORL:* les amygdalites (6 cas) et les caries dentaires (6 cas) dans la survenue des cellulites et des phlegmons periamygdaliens; l'immunodépression : (malnutrition 2 cas, VIH 1 cas); le bas niveau socio-économique: 82% de nos patients sont considérés Comme de bas niveau socio-économique.

*Traitements inappropriés:* antibiotiques: 10 patients en ont reçu avant l'hospitalisation ; anti-inflammatoires: 17 patients; traitement de médecine traditionnelle (scarification): 3 cas.

### Données cliniques et paracliniques

**Figure 1:**
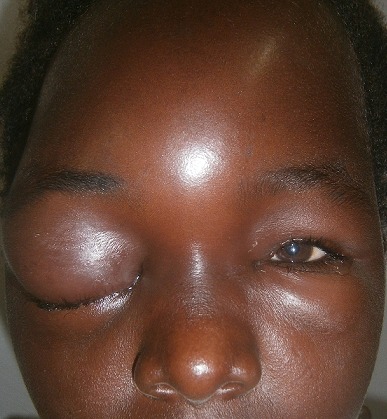
Histogramme des âges de nos patients

Le délai moyen de consultation a été de 13 jours avec des extrêmes de 4 heures et de 90 jours. Les motifs de consultations ont été variables en fonction du siège de l'infection cependant deux signes cliniques ont été constamment retrouvé : la douleur locale et la fièvre. Dans les infections de la cavité buccale et du pharynx (12 cas) les signes cliniques d'appels ont été (n = nombre de fois) : L'odynophagie (8n), la dysphagie ou l'aphagie (7n), le trismus (3n), la dyspnée haute (2n), l'otalgie reflexe (2n) et l'hypersialorrhée (1n). L'examen physique a permis dans 8 cas de poser le diagnostic. Parfois une laryngoscopie directe a été nécessaire (4 cas). Il s'est agi de 6 cas de phlegmon periamygdalien, de 4 cas d'abcès rétropharyngé et de 2 cas d'angine du plancher buccal. Dans les infections laryngées, la dyspnée laryngée a été le maître symptôme (3n) parfois accompagnée de dysphonie (2n), d'odynophagie (1n). La laryngoscopie directe a permis de confirmer leur diagnostic dans 100% de ces cas. Nous avons ainsi retrouvé 2 cas de laryngite aigue et 1 cas d'épiglottite. Les notions de douleur faciale (2n), de tuméfaction canthale et peri-orbitaire (1n) ou fronto-orbitaire (1n) ont été les signes cliniques d'appel de nos urgences rhino-sinusiennes. Suspecté à l'examen physique, la tomodensitométrie (100% des cas) a permis de confirmer l'abcès et de préciser les complications. Nous avons ainsi retrouvé 2 cas de pyocèle du sinus frontal (Figure 1), 1 cas d'abcès de la cloison nasale, 1 cas d'ethmoïdite aiguë. Les périchondrites aigues 4 cas, les mastoïdites aigues 3 cas et l'otite externe maligne 1 cas, ont été nos cas d'urgences otologiques se manifestant cliniquement par une douleur locale associée dans notre étude à une otorrhée purulente. Leur diagnostic positif a été clinique la bactériologie nous a permis d'identifier les germes en cause.

Dans les infections cervicales (25 cas), à la masse cervicale douloureuse augmentant progressivement de volume (100% des cas), s'est parfois associés une odynophagie (5 cas), une dysphagie (2 cas) et une dysphonie (1 cas). En plus de l'examen physique l'échographie cervicale réalisée chez 14 patients (56%) et la tomodensitométrie cervicale chez 1 patient (4%) nous ont permis de faire le diagnostic positif de ces urgences. Il s'est agi de 11 cas d'adénophlegmon, de 9 cas de cellulite cervicale et 5 cas de strumite. Le prélèvement micro bactériologique a été réalisé dans 46 cas (88,46%) cependant seulement 21 (45,65%) résultats bactériologiques ont été enregistrés. Les germes retrouvés sont tous aérobies. L'infection à streptocoque a dominé le tableau (8 cas), suivi des infections à staphylocoques (5 cas) à *Proteus mirabilis* (3 cas), à *Escherichia coli* (2 cas), à *Pseudomonas sp* (2 cas), et à *Klebsiela pneumonia* (1 cas). Dans 5 cas, l'étiologie a été indéterminée. Ces infections ont eu un profil monomicrobien dans 16 cas et polymicrobien dans 3 cas.

### Données thérapeutiques

Le traitement a été médico-chirurgical dans 46 cas (88,46%) et médical isolé dans 6 cas (11,54%). Au plan médical l'antibiothérapie a été instaurée de manière systématique. Les antibiotiques les plus utilisés ont été : Céphalosporines de 3^ème^ génération (45 fois soit 60% des prescriptions) associés ou non à un imidazolé (16 fois soit 21,33% des prescriptions) et ou à un aminoside (7 fois soit 9,33% des prescriptions) ; -Amoxicilline/acide clavulanique (7 fois soit 9,33% des prescriptions).

Outre l'antibiothérapie, les antalgiques ont été systématiquement prescrits et 82,69 de nos patients (43 cas) ont bénéficié d'une corticothérapie par voie générale. L'incision drainage de la collection a été pratiquée chez 46 patients. Elle a été réalisée sous anesthésie locale dans 29 cas (63%) et sous anesthésie général dans 17 cas (37%). Les patients présentant un phlegmon amygdalien ont fait l'objet d'une amygdalectomie 3 semaines à 1 mois après le geste chirurgicale, ceux présentant une carie dentaire (6 cas) ont bénéficié de soins dentaires en odontologie.

### Données évolutives

Elle a été favorable chez 42 patients (80,76%). Les complications retrouvées ont été à type de nécrose cutanée (5 cas), de choc septique (3 cas) et de méningite otogène (1 cas). Nous avons eu à noter dans notre étude 3 décès.

## Discussion

La fréquence des urgences infectieuses ORL dans notre contexte est difficilement évaluable en raison de l'absence d'étude à grande échelle. Dans la littérature cette fréquence varie d'une série à l'autre. Si pour Peres [4] ces infections sont la première cause des urgences ORL, Ramarozatovo [5] les classe en deuxième position et Bouchareb [6] en troisième position. Cependant tous ces auteurs sont unanimes sur la gravité extrême de ces urgences. Ces urgences sont l'apanage du jeune enfant et du vieillard en raison de la faiblesse de leur système immunitaire [3, 5, 7]. L'analyse du statut professionnel de nos patients a montré que 80 % d'entre eux étaient sans emploi ou avaient un emploi précaire. Ce constat est également fait par certains auteurs du nord [8, 9] qui ont noté une forte prévalence de ces infections chez les patients démunis (sans domicile fixe, sans emploi).

Dans la littérature de nombreux facteurs ont été décrits comme favorisant. C'est le cas du jeune âge, du bas niveau socio-économique, de certaines infections comme les caries dentaires et les amygdalites, de l'immunodépression (malnutrition, diabète, VIH) et de certains traitements inappropriés comme la prise d'anti-inflammatoire [10–12]. A ces facteurs il serait judicieux d'y adjoindre dans notre contexte les traitements traditionnels comme les scarifications. Le diagnostic positif de ces infections est relativement aisé. L'interrogatoire permet de retrouver des signes cliniques d'orientation tel la douleur locale et la fièvre. L'examen ORL bien mené permet de découvrir la lésion et de préciser son aspect. Parfois certaines explorations paracliniques peuvent être nécessaires. C'est le cas de la laryngoscopie directe dans les infections pharyngo-laryngées, de l'échographie de la tomodensitométrie dans les infections cervicales, nasosinusiennes, otologique et de l'analyse cytobactériologique du pus de ponction [7, 13]. Le faible pouvoir d'achat de nos patients a parfois limité la réalisation de ces examens. Les infections cervicales ou pharyngées sont décrites comme prédominantes. Elles seraient les premières manifestations cliniques des infections des éléments de de l'anneau de Waldeyer qui est le premier système de défense mise en œuvre par l'organisme en cas d'infections ORL [5, 13].

La prise en charge thérapeutique de ces infections est médico-chirurgicale. Le traitement médical repose sur une antibiothérapie initialement probabiliste instaurée dès l'admission du patient et secondairement adaptée aux résultats de l'antibiogramme [14]. Le choix de l'antibiothérapie probabiliste varie d'un auteur à l'autre. Ainsi, il n'est pas rare dans la littérature de retrouver une multitude de propositions en fonction de la pratique de chaque auteurs [1, 10, 14, 15]. Pour nous, une condition essentielle doit être respectée par l'antibiothérapie probabiliste : elle doit comporter des antibiotiques efficaces à la fois sur les aérobies et sur les anaérobies bien que dans notre contexte la mise en évidences à l'examen bactériologique des anaérobies ne soit pas aisée en raison des conditions techniques inappropriées (insuffisance du plateau technique).

Le Drainage chirurgical n'est indiqué qu'en cas d'abcès collecté. Elle peut se faire par voie cervico-faciale, endo-buccale, endoscopique. Malgré la précocité d'un traitement rigoureusement conduit après l'admission de nos patients en unité de soins intensifs, l'évolution dans notre série a été marquée par la survenue de complications. Ces complications sont essentiellement le corollaire des consultations tardives de nos patients à des stades très évolué des infections et également le fait de traitement initial non adaptées prescrits aux échelons inférieurs de la pyramide des soins. Pour McHenry [15] le retard à la consultation est le principal facteur de risque indépendant de mortalité.

## Conclusion

Les urgences infectieuses en ORL sont encore de nos jours l'une des causes les plus importantes de mortalité et de morbidité, ces urgences sont dominées par les infections cervicales, pharyngés et otologiques. De diagnostic positif relativement aisé, la prise en charge de ces urgences s'est nettement améliorée. Cependant, force est de reconnaitre que l'évolution clinique de ces infections dans notre contexte est encore émaillée de complications graves conduisant parfois au décès des patients en raison des consultations tardives.

### Etat des connaissances actuelles sur le sujet

- Fréquence selon l'âge des patients: ces infections se rencontre préférentiellement chez l'enfant de moins de 5 ans en raison de l'immaturité de son système immunitaire;
- Etiologies: fréquence élevée des infections laryngées comme les laryngites aiguées et amygdaliennes comme le Phlegmon amygdalien;
- Germes en cause: les infections sont mono ou polymicrobiennes avec des germes qui appartiennent à la fois à famille des aérobies et anaérobies.

### Contribution de notre étude à la connaissance

- Fréquence selon l'âge : Notre étude met en évidence deux pics de fréquence : chez l'enfant de moins de 10 ans et chez l'adulte de plus de 60 ans. Du fait d'une part du déficit immunitaire propre à ces deux tranche d'âge et d'autre part de l'apparition de certains facteurs favorisants comme le diabète propre au sujet âgé;
- Etiologies: les étiologies les plus fréquentes dans notre série sont cervico-faciales : abcès ganglionnaire (adénophlegmon) et les cellulites en raison des consultations tardive de nos patients au stade compliqués d'infection ORL et d'odontostomatologie classiques comme les adénites, les amygdalites, les caries dentaires;
- Germes en causes: dans notre série les résultats des examens bactériologiques ont retrouvées des infections mono microbiennes avec des germes de la famille des aérobies. Nous estimons que nos résultats bactériologiques sont le fait de notre plateau technique inapproprié à la mise en évidence des anaérobies et nous estimons que malgré ces résultats, l'antibiothérapie probabiliste doit comporter une condition essentielle : être a la fois efficace contre les aérobies et les anaérobies.
